# Association between air pollution exposure and mental health service use among individuals with first presentations of psychotic and mood disorders: retrospective cohort study

**DOI:** 10.1192/bjp.2021.119

**Published:** 2021-12

**Authors:** Joanne B. Newbury, Robert Stewart, Helen L. Fisher, Sean Beevers, David Dajnak, Matthew Broadbent, Megan Pritchard, Narushige Shiode, Margaret Heslin, Ryan Hammoud, Matthew Hotopf, Stephani L. Hatch, Ian S. Mudway, Ioannis Bakolis

**Affiliations:** Centre for Academic Mental Health and MRC Integrative Epidemiology Unit, Population Health Sciences, Bristol Medical School, University of Bristol; and King's College London, Social, Genetic & Developmental Psychiatry Centre, Institute of Psychiatry, Psychology & Neuroscience, London, UK; King's College London, Psychological Medicine, Institute of Psychiatry, Psychology & Neuroscience, London; and South London and Maudsley NHS Foundation Trust, London, UK; King's College London, Social, Genetic & Developmental Psychiatry Centre, Institute of Psychiatry, Psychology & Neuroscience, London; and ESRC Centre for Society and Mental Health, King's College London, UK; Environmental Research Group, School of Public Health, Faculty of Medicine, Imperial College London; and MRC Centre for Environment and Health, School of Public Health, Faculty of Medicine, Imperial College London, UK; King's College London, Psychological Medicine, Institute of Psychiatry, Psychology & Neuroscience, London; and NIHR Biomedical Research Centre, South London and Maudsley NHS Foundation Trust, London, UK; Department of Geography, King's College London, UK; King's College London, King's Health Economics, Institute of Psychiatry, Psychology & Neuroscience, London, UK; King's College London, Department of Psychosis Studies, Division of Academic Psychiatry, Institute of Psychiatry, Psychology and Neuroscience, London, UK; King's College London, Psychological Medicine, Institute of Psychiatry, Psychology & Neuroscience, London; and ESRC Centre for Society and Mental Health, King's College London, UK; Environmental Research Group, School of Public Health, Faculty of Medicine, Imperial College London; and MRC Centre for Environment and Health, School of Public Health, Faculty of Medicine, Imperial College London; and NIHR Health Protection Research Unit in Environmental Exposures and Health, School of Public Health, Faculty of Medicine, Imperial College London, UK; King's College London, Centre for Implementation Science, Health Services and Population Research Department, Institute of Psychiatry, Psychology and Neuroscience, London; and King's College London, Department of Biostatistics and Health Informatics, Institute of Psychiatry, Psychology and Neuroscience, London, UK

**Keywords:** Air pollution, psychotic disorders, mood disorders, cohort study, illness severity and relapse

## Abstract

**Background:**

Growing evidence suggests that air pollution exposure may adversely affect the brain and increase risk for psychiatric disorders such as schizophrenia and depression. However, little is known about the potential role of air pollution in severity and relapse following illness onset.

**Aims:**

To examine the longitudinal association between residential air pollution exposure and mental health service use (an indicator of illness severity and relapse) among individuals with first presentations of psychotic and mood disorders.

**Method:**

We identified individuals aged ≥15 years who had first contact with the South London and Maudsley NHS Foundation Trust for psychotic and mood disorders in 2008–2012 (*n* = 13 887). High-resolution (20 × 20 m) estimates of nitrogen dioxide (NO_2_), nitrogen oxides (NO_x_) and particulate matter (PM_2.5_ and PM_10_) levels in ambient air were linked to residential addresses. In-patient days and community mental health service (CMHS) events were recorded over 1-year and 7-year follow-up periods.

**Results:**

Following covariate adjustment, interquartile range increases in NO_2_, NO_x_ and PM_2.5_ were associated with 18% (95% CI 5–34%), 18% (95% CI 5–34%) and 11% (95% CI 3–19%) increased risk for in-patient days after 1 year. Similarly, interquartile range increases in NO_2_, NO_x_, PM_2.5_ and PM_10_ were associated with 32% (95% CI 25–38%), 31% (95% CI 24–37%), 7% (95% CI 4–11%) and 9% (95% CI 5–14%) increased risk for CMHS events after 1 year. Associations persisted after 7 years.

**Conclusions:**

Residential air pollution exposure is associated with increased mental health service use among people recently diagnosed with psychotic and mood disorders. Assuming causality, interventions to reduce air pollution exposure could improve mental health prognoses and reduce healthcare costs.

Psychotic and mood disorders have a lifetime prevalence of around 3%^1^ and 17%^2^ respectively, with each contributing substantially to the global burden of disease.^3^ Considerable heterogeneity exists in the course of these psychiatric conditions following onset, ranging from single and brief episodes for some individuals to repeated relapse and a chronic course for others.^4,5^ Identifying modifiable risk factors for illness severity and relapse following onset is therefore a crucial research challenge that could inform early-intervention efforts and reduce the human suffering and high economic costs caused by long-term chronic mental illness. One potential modifiable risk factor is ambient air pollution, which is estimated to cause 482 000 premature deaths per year in Europe alone, at a cost of $1.575 trillion.^6^ These estimates are based on impacts on cardiorespiratory diseases, but emerging evidence suggests that air pollution can also adversely affect the brain^7^ and increase risk for psychiatric disorders such as schizophrenia^8^ and depression.^9^ However, very little is known about the potential role of air pollution exposure in illness severity and relapse following first presentation for psychotic or mood disorder. Furthermore, existing studies have often been cross-sectional or short term, relied on aggregated pollution and mental health data, and have lacked appropriate adjustment for potential confounders.

Thus, we aimed to examine the longitudinal association between residential air pollution exposure and mental health service use after first presentation for psychotic or mood disorder. Our outcome – duration and frequency of mental health service use – provides a marker of illness severity and relapse in the context of psychiatric disorders,^10,11^ because those with persistent symptoms and/or multiple episodes are likely to require more frequent interaction with services over a longer period of time. We used a state-of-the-art air quality model to estimate address-level air pollution exposure in four highly urbanised and ethnically diverse South London boroughs, and considered numerous potential individual- and area-level confounders. Comprehensive electronic records of mental health service use were recorded for up to 7 years. Our main hypothesis was that higher air pollution exposure around the time of first presentation contributes to increased mental health service use, in both the short term (over the first year) and longer term (over 7 years).

## Method

### Study design and participants

**Fig. 1:**
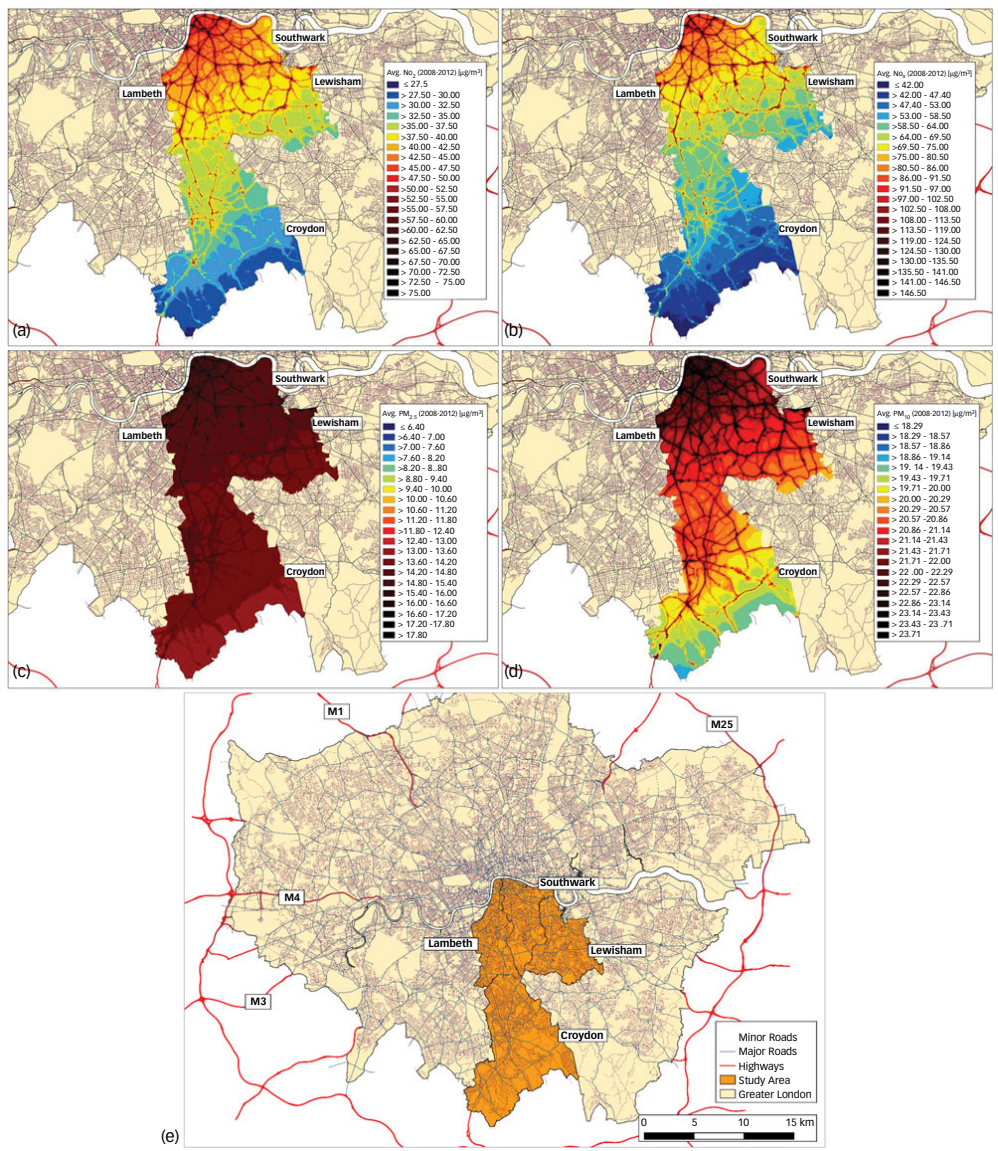
Air pollution concentrations in the four-borough catchment area averaged across 2008–2012. (a) Nitrogen dioxide (NO_2_). (b) Nitrogen oxides (NO_x_). (c) Particulate matter <2.5 μm in diameter (PM_2.5_). (d) Particulate matter <10 μm in diameter (PM_10_). (e) The four-borough catchment area in Greater London. World Health Organization recommended annual mean air quality limits for human health: NO_2_, 40 μg/m^3^; PM_2.5_, 10 μg/m^3^; PM_10_, 20 μg/m^3^. European Union annual mean air quality limits for ecosystems and vegetation: NO_x_, 30 μg/m_3_.

We conducted a retrospective cohort study. Supplementary Fig. 1, available at https://doi.org/10.1192/bjp.2021.119, illustrates the timeline of entry into the cohort and collection of measures. The South London and Maudsley National Health Service (NHS) Foundation Trust (SLaM) is one of Europe's largest secondary mental healthcare providers.^12^ The trust provides comprehensive secondary mental healthcare to a catchment area of approximately 1.36 million people within the London boroughs of Southwark, Lambeth, Lewisham and Croydon (Fig. 1).

Since 2006, SLaM has operated fully electronic health records. The Clinical Record Interactive Search (CRIS) system, established in 2008, is an ethically approved platform and governance framework that allows researchers to access complete, de-identified data from trust records for research.^12^ CRIS holds all information documented by professionals involved in the provision of specialist mental healthcare for all people in contact with SLaM services from 1 January 2007 to date, in addition to earlier legacy data.^12,13^ CRIS has received ethical approval as an anonymised data resource for secondary analyses (Oxford Research Ethics Committee C, reference 18/SC/0372).

All clinicians and mental health teams are required to assign ICD-10 psychiatric diagnoses to individuals under the care of SLaM. Individuals with ICD-10 diagnoses of schizophrenia-spectrum disorders (F2*) and mood disorders (F3*) were identified through searches of CRIS-structured fields, supplemented by a natural language processing application developed using General Architecture for Text Engineering (GATE), which extracts diagnostic statements from the free text of case notes and clinical correspondence.^12,14^ Our sample included individuals aged 15 years and over who had a first face-to-face contact in SLaM between 1 January 2008 and 31 December 2012 and were given a primary diagnosis of any schizophrenia, schizotypal or delusional disorders (F20–F29) or any mood or affective disorders (F30–F39).^15^ Individuals were followed from the date of their first face-to-face contact (baseline) for up to 7 years. Our data-extraction frame accounted for the co-occurrence and heterotypical continuity often found between psychotic and mood disorders: for instance, individuals could have received secondary psychiatric diagnoses at first presentation and over the course of follow-up. Air pollution was modelled for Southwark, Lambeth, Lewisham and Croydon (Fig. 1). Therefore, we only included individuals who resided within these four boroughs at the time of first face-to-face contact. Participants of no fixed abode were also excluded by design.

### Measures

Mental health service use was measured in two ways to capture different pathways to care, and over 1- and 7-year follow-up periods following first face-to-face contact to explore both shorter- and longer-term associations. First, in-patient days were estimated as the number of in-patient bed-days and home treatment team (HTT: a home-based alternative to in-patient care) days over 1 year and 7 years. These were calculated using dates of admissions and discharges from in-patient wards and HTT services. Second, community mental health service (CMHS) events were estimated as the number of distinct face-to-face attended appointments, including, for example, out-patient visits and appointments with specialist teams (but excluding HTT visits) over 1 year and 7 years. Since our sample included teenagers, in-patient days and CMHS events included service use occurring within child and adolescent mental health services (CAMHS). The total time under the care of SLaM was calculated as the time between the first face-to-face contact and referral discharge within the 1- and 7-year follow-up periods.

High-resolution (20 × 20 m) air pollution models for Greater London were produced for 2008 to 2012 using KCLurban, based on the Atmospheric Dispersion Modelling System model v4 and Road Source model v2.3 (Cambridge Environmental Research Consultants), hourly meteorological data, empirically derived atmospheric pollutant relationships and emission estimates recorded in the London Atmospheric Emissions Inventory. Exposure data were outputted as quarterly (3-month) mean concentrations of nitrogen dioxide (NO_2_), nitrogen oxides (NO_x_), particulate matter with an aerodynamic diameter <2.5 μm (PM_2.5_) and particulate matter with an aerodynamic diameter <10 μm (PM_10_). Quarterly outputs covered the 3-month period in which the first face-to-face contact took place. We hypothesised that air pollution exposure during this time might be particularly relevant to subsequent illness trajectories, given that air pollutants have potent inflammatory and oxidative properties, and given evidence that inflammation and oxidative stress appear to contribute to the onset of psychotic and mood disorders.^16,17^ As a de-identified data resource, CRIS holds no address data on patients. Therefore, linkage of patients’ addresses to air pollution data was undertaken within a pre-CRIS data processing pipeline. Pollution exposure estimates used the bilinear interpolation method using the four 20 × 20 m points around each address. A comprehensive description of the model and its validation against ground-based measurements has been published previously.^18^ For instance, the model's predictions perform well against actual observations, with correlations in 2008 exceeding *r*^2^ = 0.93.^18^

### Confounders

Quarter and year of first face-to-face contact were included as covariates to control for seasonal fluctuations and annual trends in air pollution concentrations and psychiatric admissions. Individual-level covariates included gender and ethnicity, as well as age and marital status at initial presentation, which were extracted from structured data. Ethnicity was based on ethnicity categories in the 2001 UK census. Area-level covariates included population density, deprivation, ethnic density and social fragmentation, which were linked to residential addresses at first face-to-face contact (detailed further in Heslin et al^10^ and the supplementary materials). Population density was determined using 2011 census data based on number of people per hectare. Neighbourhood deprivation was classified using 2011 Index of Multiple Deprivation (IMD) scores at the lower-layer super output area (LSOA) level, which includes on average 1500 residents. Ethnic density was defined as the proportion of people from the same ethnic group as the participant living in their LSOA, also estimated from the 2011 census. Social fragmentation was defined at the LSOA level as a *z*-scored composite of four measures from the 2011 census: unmarried adults, single-person households, households privately renting and population turnover.

### Statistical analyses

Data analyses were performed using Stata v14.2. Sample characteristics were described using percentages, means and standard deviations. For analysis, air pollutants were rescaled to interquartile range (IQR) increments as well as increments specific to the quartile distribution of each pollutant. Rescaling to the IQR allows effect estimates to be calculated for comparable increases across different pollutants, which may have very different absolute concentration ranges. Associations between air pollution exposure and in-patient days and CMHS events were explored using zero-inflated and standard negative binomial regression models respectively. Time in contact with services was treated as the offset variable, to account for differences in follow-up period (e.g. owing to moving out of the catchment area). All models were fitted separately for each outcome and air pollutant, and adjusted for seasonality and year (model 1), plus gender, ethnicity, age and marital status (model 2), plus population density, deprivation, ethnic density and social fragmentation (model 3). All models only included cases who had complete data in model 3. As recommended for observational research,^19^ we also calculated E-values, which represent the strength of association that an unmeasured confounder would require with the exposure and outcome to attenuate main associations to non-significance. To estimate the percentage of mental health service use that could be attributable to air pollution, we then calculated population attributable fractions (PAFs) for two exposure scenarios (our four South London boroughs and UK urban areas). We purposely selected PM_2.5_ to calculate PAFs because mean concentrations of PM_2.5_ in our study exceeded the World Health Organization's (WHO's) recommended annualised mean limit (10.0 μg/m^3^) by a greater margin than the other air pollutants, thereby providing the clearest counterfactual scenario. Further details on PAF calculations are provided in the supplementary materials. Finally, we ran seven sensitivity analyses:

- to explore specificity of psychiatric diagnosis we conducted subgroup analyses according to psychotic disorder diagnoses versus mood disorder diagnoses;
- to address biases due to missing covariate data we repeated analyses following multiple imputation by chained equations (detailed further in the supplementary materials);
- to examine whether associations were modified by neighbourhood deprivation we included an interaction term with IMD quartiles in the regression models;
- to examine whether associations varied significantly by borough of residence we included an interaction term with borough in the regression models;
- to examine co-pollutant confounding, we ran two-pollutant models in which each pollutant was included as a covariate with every other pollutant;
- to address misclassification and uncertainties over diagnosis we restricted analyses to participants who were diagnosed within 30 days of first contact;
- to address the problem of residential mobility across follow-up, we restricted analyses to participants who were living at the same address (i) 3 months, (ii) 1 year and (iii) 7 years after first contact with SLaM; for (ii) and (iii), we also used annualised rather than quarterly pollution data, given that restricting to non-movers minimised potential exposure misclassification.

## Results

### Descriptive analysis

A total of 19 545 people aged ≥15 years had first face-to-face contact with SLaM services between 1 January 2008 and 31 December 2012 and received a diagnosis of psychotic (F20–F29) or mood (F30–F39) disorder. Of these people, 3508 were living outside the catchment area and 2150 could not be linked to the air quality model and were excluded from analyses. The eligible sample therefore included 13 887 individuals. Table 1 describes data for this sample and supplementary Fig. 2 presents a flow diagram of the eligible and complete case sample. Mean age at first face-to-face contact was 41.6 years, 58.8% of the sample were single, 58.7% were female and 46.3% were of White British ethnicity.

**Table 1:** Sample characteristics and air pollution exposures

Covariates		
Individual-level covariates		
Age, years: mean (s.d.)	41.6	(19.4)
Gender, *n* (%)		
Female	8151	(58.7)
Male	5736	(41.3)
Ethnicity, *n* (%)		
Any other Asian background	351	(2.6)
Any other Black background	1102	(8.1)
Any other ethnic group	1388	(10.3)
Any other mixed background	104	(0.8)
Any other White background	1289	(9.5)
Bangladeshi	63	(0.5)
Black African	1234	(9.1)
Black Caribbean	788	(5.8)
Chinese	86	(0.6)
Indian	174	(1.3)
Irish	320	(2.4)
Pakistani	116	(0.9)
White and Asian	25	(0.2)
White and Black African	57	(0.4)
White and Black Caribbean	172	(1.3)
White British	6269	(46.3)
Marital status, *n* (%)		
Cohabiting	368	(2.8)
Divorced	287	(2.2)
Divorced/civil partnership dissolved	434	(3.3)
Married	1058	(8.1)
Married/civil partner	1596	(12.3)
Separated	665	(5.1)
Single	7640	(58.8)
Widowed	378	(2.9)
Widowed/surviving civil partner	575	(4.4)
Area-level covariates, mean (s.d.)^a^		
Ethnic density	0.3	(0.2)
Neighbourhood deprivation	30.1	(9.7)
Population density	108.6	(52.7)
Social fragmentation	3.2	(2.1)
Air pollution exposure, mean (s.d.), μg/m^3^^b^		
NO_2_	40.5	(10.1)
NO_x_	71.7	(26.2)
PM_2.5_	14.5	(2.9)
PM_10_	21.6	(4.2)

NO_2_, nitrogen dioxide; NO_x_, nitrogen oxides; PM_2.5_, particulate matter with a diameter of <2.5 μm; PM_10_, particulate matter with a diameter of <10 μm.

a. Units for area-level covariates: ethnic density, proportion of people residing in the same lower-layer super output area as the participant with the same ethnicity as the participant; neighbourhood deprivation, Index of Multiple Deprivation score; population density, persons per hectare; social fragmentation, *z*-scored composite of unmarried adults, single-person households, housing tenure and population turnover.

b. Units for air pollution exposure: μg/m^3^.

At 1-year and 7-year follow-up, mean time under the care of SLaM was 211 days and 691 days respectively, ranging from 1 day to the full 1 or 7 years. Mean number of in-patient days after 1 year and 7 years was 14 days (range 0–366) and 36 days (range 0–2556) respectively. Mean number of CMHS events after 1 year and 7 years was 11 events (range 1–272) and 33 events (range 1–1026) respectively. Of the total sample, 23% (*n* = 3132) had a primary psychotic disorder diagnosis (of whom 58 had a secondary mood disorder diagnosis) and 77% (*n* = 10 755) had a primary mood disorder diagnosis (of whom 224 had a secondary psychotic disorder diagnosis). Figure 1 presents maps of air pollution concentrations in the catchment area, averaged across 2008–2012.

### Association between air pollution and mental health service use

**Fig. 2:**
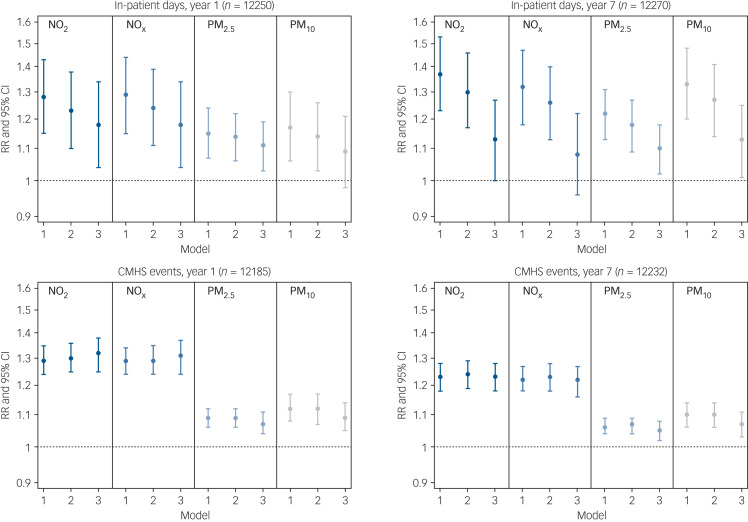
Associations between interquartile range increases in air pollution exposure and mental health service use over 1-year and 7-year follow-up. CMHS, community mental health services; NO_2_, nitrogen dioxide; NO_x_, nitrogen oxides; PM_2.5_, particulate matter <2.5 μm in diameter; PM_10_, particulate matter <10 μm in diameter; RR, relative risk. Model 1 was adjusted for seasonality and year. Model 2 was adjusted additionally for gender, ethnicity, age and marital status. Model 3 was adjusted additionally for population density, deprivation, ethnic density and social fragmentation.

Figure 2 displays relative risks and confidence intervals for the association between estimated air pollution exposure and mental health service use, presented according to 1-year and 7-year follow-up periods. Supplementary Table 1 presents the exact values together with E-values. Higher exposure to NO_2_, NO_x_, PM_2.5_ and PM_10_ was associated with more in-patient days and CMHS events across follow-up. After full covariate adjustment (model 3 in Fig. 2), IQR increases in NO_2_, NO_x_ and PM_2.5_ were significantly associated with 18% (95% CI 4–34%), 18% (95% CI 4–34%) and 11% (95% CI 3–19%) risk increases in in-patient days respectively at 1-year follow-up. Furthermore, IQR increases in NO_2_, NO_x_, PM_2.5_ and PM_10_ were significantly associated with 32% (95% CI 25–38%), 31% (95% CI 24–37%), 7% (95% CI 4–11%) and 9% (95% CI 5–14%) risk increases in CMHS events respectively at 1-year follow-up. Thus, although of a comparable magnitude, associations were more robust for CMHS events than for in-patient days in terms of the precision of confidence intervals and resistance to covariate adjustment. Associations were also strongest in magnitude for NO_2_ and NO_x_ and persisted at 7-year follow-up (Fig. 2).

Table 2 presents associations across quartile increments for each air pollutant. There was evidence of a dose–response relationship within the associations observed, with incremental risk increases across quartiles of exposure. For instance, participants in the second, third and fourth quartiles (versus the first quartile) of NO_2_ exposure had a 16% (95% CI 10–23%), 32% (95% CI 22–42%) and 48% (95% CI 36–62%) increased risk for CMHS events respectively after 1 year.

**Table 2:** Association between quartiles of air pollution exposure (Q1–Q4) and mental health service use over 1-year and 7-year follow-up^a^

Outcome and pollutant	1-year follow-up						7-year follow-up					
	Complete case sample, *n*	Quartiles of air pollution exposure					Complete case sample, *n*	Quartiles of air pollution exposure				
		Q1 (ref)	Q2, RR (95% CI)	Q3, RR (95% CI)	Q4, RR (95% CI)	E-value, RR (lower CI)		Q1 (ref)	Q2, RR (95% CI)	Q3, RR (95% CI)	Q4, RR (95% CI)	E-value, RR (lower CI)
In-patient days	12 250						12.270					
NO_2_		−	1.32*** (1.16−1.51)	1.21* (1.01−1.46)	1.46*** (1.18−1.80)	2.28 (1.64)		−	1.49*** (1.30−1.70)	1.27* (1.06−1.53)	1.22 (0.99−1.51)	1.74 (1.00)
NO_x_		−	1.33*** (1.16−1.52)	1.22* (1.00−1.47)	1.45*** (1.17−1.81)	2.26 (1.62)		−	1.46*** (1.27−1.67)	1.26* (1.04−1.51)	1.17 (0.94−1.44)	1.62 (1.00)
PM_2.5_		−	1.22* (1.01−1.47)	1.32* (1.06−1.65)	1.25 (0.97−1.60)	1.81 (1.00)		−	0.96 (0.80−1.16)	0.97 (0.78−1.21)	1.28* (1.00−1.63)	1.88 (1.00)
PM_10_		−	1.06 (0.92−1.23)	0.70*** (0.58−0.85)	0.93 (0.75−1.14)	1.36 (1.00)		−	0.91 (0.79−1.05)	0.77* (0.64−0.93)	1.16 (0.94−1.43)	1.59 (1.00)
CMHS events	12 185						12 232					
NO_2_		−	1.16*** (1.10−1.23)	1.32*** (1.23−1.42)	1.48*** (1.36−1.62)	2.32 (2.06)		−	1.14*** (1.08−1.19)	1.26*** 1.18−1.36)	1.36*** (1.26−1.47)	2.06 (1.83)
NO_x_		−	1.17*** (1.11−1.23)	1.32*** (1.22−1.42)	1.49*** (1.37−1.63)	2.34 (2.08)		−	1.13*** (1.08−1.19)	1.24*** (1.16−1.32)	1.34*** (1.24−1.45)	2.01 (1.79)
PM_2.5_		−	1.18*** (1.10−1.27)	1.19*** (1.09−1.30)	1.24*** (1.13−1.36)	1.79 (1.51)		−	1.12** (1.05−1.19)	1.11** (1.03−1.19)	1.15** (1.06−1.25)	1.57 (1.31)
PM_10_		−	1.01 (0.95−1.07)	1.02 (0.94−1.10)	1.12* (1.03−1.22)	1.49 (1.21)		−	0.96 (0.91−1.01)	1.04 (0.97−1.11)	1.09* (1.01−1.17)	1.40 (1.11)

CMHS, community mental health services; E-value, association required between unmeasured confounder(s) and both the exposure and outcome to make Q4 effects non-significant, above and beyond measured covariates; NO_2_, nitrogen dioxide; NO_x_, nitrogen oxides; PM_2.5_, particulate matter <2.5 μm in diameter; PM_10_, particulate matter <10 μm in diameter; RR, relative risk.

a. The quartile cut-offs for NO_2_ were: Q1 <32.65 μg/m^3^, Q2 <39.41 μg/m^3^, Q3 <48.08 μg/m^3^ and Q4 ≥48.08 μg/m^3^; for NO_x_ they were: Q1 <50.74 μg/m^3^, Q2 <66.83 μg/m^3^, Q3 <90.50 μg/m^3^ and Q4 ≥90.50 μg/m^3^; for PM_2.5_ they were: Q1 <12.64 μg/m^3^, Q2 <14.28 μg/m^3^, Q3 <15.36 μg/m^3^ and Q4 ≥15.36 μg/m^3^; and for PM_10_ they were: Q1 <18.81 μg/m^3^, Q2 <21.41 μg/m^3^, Q3 <24.50 μg/m^3^ and Q4 ≥24.50 μg/m^3^. All models were adjusted for seasonality, year, gender, ethnicity, age, marital status, population density, deprivation, ethnic density and social fragmentation. Analyses were conducted on those with complete covariate data.

* *P* < 0.05, **P < 0.01, ***P < 0.001.

Table 2 and supplementary Table 1 also include E-values, which indicate robustness to unmeasured confounding. E-values were large relative to point estimates for the included covariates. We illustrate this in supplementary Table 2 for the association between NO_2_ and in-patient days by comparing covariate point estimates with E-values. This increases our confidence in the results, because it suggests that any unmeasured confounder(s) would require a stronger association with the exposure and outcome than was found for all covariates included in our analyses in order to attenuate main associations to non-significance.

### Sensitivity analyses

Results from the seven sensitivity analyses are as follows and are interpreted in detail in the Discussion. (a) In terms of diagnosis specificity, associations were often slightly stronger in magnitude for mood compared with psychotic disorders, although confidence intervals overlapped (supplementary Table 3). (b) Results following multiple imputation were consistent with the original complete case associations (supplementary Table 4). (c) There was little evidence that associations were modified by deprivation level, except in models for NO_2_, NO_x_ and CMHS events, in which case a negative interaction was observed (supplementary Table 5). (d) There was also little evidence that associations were modified by borough of residence, except for Croydon, in which case interactions varied in direction depending on air pollutant (supplementary Table 6). (e) In two-pollutant models, associations often changed substantially in magnitude and direction (supplementary Table 7). (f) When examining diagnosis misclassification, effects were similar though slightly stronger than the original associations (supplementary Table 8). (g) Finally, when examining residential mobility, associations mostly persisted for those living at the same address 3 months (supplementary Table 9), 1 year (supplementary Table 10) and 7 years (supplementary Table 11) after first presentation, although point estimates often increased somewhat for PM_2.5_ and PM_10_.

### Population attributable fractions

Our analysis suggested that if the population-weighted PM_2.5_ exposure (13.4 μg/m^3^ in 2019) in our four South London boroughs was reduced by 3.4 μg/m^3^ to the WHO's recommended annual limit (10 μg/m^3^), then annual in-patient and CMHS use could be reduced by 2.9% (95% CI 0.9–5.8%) and 2.0% (95% CI 1.0–2.9%) respectively (supplementary Table 12). For other UK urban areas, in-patient and CMHS use could be reduced by approximately 2.0% (95% CI 0.9–3.8%) and 1.9% (95% CI 0.9–2.9%) respectively.

## Discussion

In a retrospective cohort of 13 887 individuals with a first presentation of psychotic or mood disorder, those with higher residential air pollution exposure used mental healthcare services more frequently in the months and years following their initial presentation to secondary mental healthcare services. These associations were comparable between in-patient and CMHS use, suggesting that air pollution may be relevant across the spectrum of clinical need. However, we noted more robust and precise associations for CMHS use than for in-patient use. This could partly be because CMHS use was more common than in-patient use. Furthermore, the pollution–in-patient association may have been subject to more confounding due to ethnic disparities in pathways to care, given that in London, Black African and Black Caribbean people are more likely to be forcibly detained under the Mental Health Act 1983 than White British people.^20^ It could also be that exposure misclassification had a greater impact on the pollution–in-patient association, given that those with higher in-patient use would have spent more time away from their home (the model's target). Nevertheless, associations were generally robust to a wide range of possible confounders and persisted over several years of follow-up. Our confidence in the associations was strengthened by the relatively large E-values, as well as the consistent results identified following several sensitivity analyses.

### Sensitivity analyses

Four sensitivity results warrant discussion. First, counterintuitively, there was tentative evidence for a negative interaction of NO_2_ and NO_x_ with deprivation, such that the association between air pollution and CMHS use appeared to be stronger in the least deprived settings. We hypothesise that this reflected our catchment area, four London boroughs in which some of the most affluent inner-city neighbourhoods also have among the busiest roads and, consequently, among the highest pollution levels. Second, associations between air pollution and mental health service use differed between Croydon and the other boroughs. In addition to having lower average pollution concentrations than the other boroughs, Croydon also has lower availability of in-patient and HTT services, and we hypothesise a role of these factors in the differences observed. Third, results from the two-pollutant model reflected the high multicollinearity between pollutants: the variance inflation factor, an indicator of multicollinearity, was >5 for all two-pollutant models. From a modelling perspective, estimates could be augmented with a multi-pollutant approach, as individual-pollutant approaches might underestimate effect sizes. Fourth, associations for PM_2.5_ and PM_10_ increased in magnitude when using annualised data and restricting to non-movers. This could suggest that exposure misclassification and/or seasonal pollution patterns had a greater impact on the association of service use with PM_2.5_ and PM_10_ than with NO_2_ and NO_x._

### Strengths and limitations

Recent time-series studies have documented associations between short-term, city-level air pollution concentrations and daily hospital admissions for schizophrenia and depression.^21,22^ We build on this research in several ways. Our air pollution data achieve high spatial precision, thereby reducing potential exposure misclassification. We also estimated illness severity and relapse using two different forms of mental health service use, and over the course of several years following first presentation. Our study population is representative of South London's ethnic diversity and broad socioeconomic spectrum. The catchment area also reflects the air pollution patterns found for the whole of London and, indeed, all large cities with heavy diesel vehicle traffic, with concentrations increasing from the outer to inner city. Other strengths include the steps taken to address potential biases, including measured and unmeasured confounding and missing data. Thus, our findings contribute novel evidence that higher air pollution exposure increases illness severity and relapse in the months and years following initial presentation for psychotic and mood disorders. Our findings also align with those from a recent study^23^ covering a similar catchment area (South East London Community Health Study: SELCoH), in which higher air pollution was associated with increased odds for subclinical psychotic symptoms and common mental disorders in the general population. Together, these findings provide converging evidence that air pollution exposure may contribute to the onset and severity of psychiatric problems, spanning subclinical through to clinical manifestations. We also acknowledge several limitations. First, mental health service use is a proxy for illness severity and relapse. Other factors could also influence duration and frequency of contact with services, such as bed availability and risk evaluations. Second, given that air pollution data spanned 2008–2012, we were unable to examine associations of early-life air pollution exposure with mental health service use. Third, our address-level resolution will nevertheless have masked changes in exposure due to residential mobility, behaviours (e.g. commuting) and time spent away from home (e.g. long periods on a psychiatric ward). Wearable monitors would be required to address such limitations, and these remain prohibitively expensive in large cohort studies. Fourth, although our findings probably generalise to cities in other high-income countries, the generalisability to low- and middle-income country (LMIC) urban settings, which often have much higher pollution levels (www.iqair.com/world-air-quality-ranking) and lower psychiatric healthcare provision, is uncertain. Fifth, the causality of the observational findings is uncertain and residual confounding is inevitable, although we calculated E-values to evaluate unmeasured confounding.

#### Mechanisms linking air pollution to psychotic and mood disorders

With potent oxidising and inflammatory properties, it has been suggested that air pollutants could affect the brain directly by translocating along the olfactory nerve and permeating the blood–brain barrier and/or indirectly by eliciting systemic inflammation.^7^ Neuroinflammation and oxidative stress are likewise both implicated in the aetiology of psychotic^16^ and mood disorders,^17^ and therefore a role of air pollution exposure in the severity and course of psychotic and mood disorders is biologically plausible. In our study, associations were strongest for NO_2_ and NO_x_, which are closely correlated with heavy traffic and diesel vehicle emissions. However, whether these associations were directly attributable to NO_2_ and NO_x_ or, rather, to correlated combustion particle emissions from diesel vehicle exhaust-pipes remains uncertain.

### Implications and future directions

If we assume causality and accept mental health service use as a proxy for illness severity and relapse, air pollution should be considered an important population-level target to improve the course of psychotic and mood disorders. Reducing air pollution exposure, for example by expanding low-emission zones in urban areas, could potentially improve outcomes for people with first presentations of psychotic and mood disorders. Alongside the human suffering caused by persistent and recurrent mental health problems are the high mental healthcare costs. In England, allowing for inflation, in-patient services and CMHS are estimated to cost respectively £507.50 million and £37.15 million per year for schizophrenia^24^ and £47.70 million and £36.84 million per year for depression.^25^ We calculated that reducing PM_2.5_ levels to WHO's recommended threshold would reduce in-patient and CMHS demand in UK urban areas by 2.0 and 1.9% respectively, thereby reducing the associated healthcare costs as well as improving service capacity and waiting times. However, there remain pressing questions around the causality of associations between air pollution and mental health. Addressing these questions will require continued longitudinal and causally informed research, especially in LMIC contexts, together with biological insights into the mechanisms linking air pollution to psychopathology.

## Data Availability

The data that support the findings of this study are not publicly available but can be accessed with permissions from the South London and Maudsley NHS Foundation Trust.

## Supplementary material

For supplementary material accompanying this paper visit http://dx.doi.org/10.1192/bjp.2021.119. click here to view supplementary material
