# Supplementary material for: Association between air pollution exposure and mental health service use among individuals with first presentations of psychotic and mood disorders: retrospective cohort study
Source: Br J Psychiatry. 2021 Dec;219(6):678–85. doi: 10.1192/bjp.2021.119 (PMC8636613; doi:10.1192/bjp.2021.119)
Supplement: Supplementary file 1 [file S0007125021001197sup001.docx]

**Supplementary Materials**

**Association of air pollution exposure with mental health service-use among individuals with first presentations of psychotic and mood disorders:**

**a retrospective cohort study**

Joanne B Newbury, Robert Stewart, Helen L Fisher, Sean Beevers, David Dajnak,

Matthew Broadbent, Megan Pritchard, Narushige Shiode, Margaret Heslin, Ryan Hammoud, Matthew Hotopf, Stephani L Hatch, Ian S Mudway, Ioannis Bakolis

**Methods**

*Individual-level covariates* included sex, ethnicity, as well as age and marital status at initial presentation, which were extracted from structured data. Ethnicity was based on ethnicity categories in the 2001 UK census and was coded for analysis such that 0=White British, 1=Irish and any other White background, 2=Black African, 3=Caribbean and any other Black background; 4=Indian, Pakistani, and Bangladeshi, and 5=White and Black Caribbean, White and Black African, White and Asian, any other mixed background, any other Asian background, Chinese, or any other ethnic group. Likewise, marital status was coded for analysis such that 0=married/civil partnership/cohabiting, 1=single, 2=divorced/civil partnership dissolved/separated, and 3=widowed/surviving civil partner.

*Area-level covariates.* Analyses were adjusted for population density, deprivation, ethnic density, and social fragmentation. As a deidentified data resource, CRIS holds no address or postcode data. Thus, linkage of service-user addresses to lower- (LSOA) and middle-layer (MSOA) super output areas was conducted within a pre-CRIS data processing pipeline. LSOAs and MSOAs are geographical areas made up of clusters of socially homogenous postcodes. LSOAs have an average of 1500 residents while MSOAs have an average of 7200 residents.^1^ Population density was determined using 2011 census data based on the number of people per hectare.^2^ Neighbourhood deprivation was classified using 2011 Index of Multiple Deprivation (IMD) scores at the LSOA-level,^3^ assigned to residential addresses at first face-to-face contact. Ethnic density was defined as the proportion of people from the same ethnic group as the participant living in their LSOA,^4^ also estimated from the 2011 census. Social fragmentation was calculated for each LSOA from four measures of social composition in the 2011 census: unmarried adults, single-person households, households privately renting, and population turnover (the sum of in- and out-migration in the 12 months prior to the 2011 census^5^ at MSOA-level). Consistent with previous studies,^6^ we summed the *z-*scores to create a social fragmentation index.

*Multiple imputation by chained equations*. Complete case analyses were conducted on those without missing data in Model 3. Between 11.6% and 12.3% (N=1,617–1,702) of the sample had missing data in Model 3. Multiple imputation by chained equations (MICE) was then performed for these missing data within Stata v14.2 (“mi impute chained”) for all patients with valid postcode information who resided in the four-borough catchment area and were aged ≥15 years at first face-to-face contact. Imputations were performed for all analysis variables with missing values, including inpatient days, community mental health services (CMHS) events, marital status, ethnicity, social fragmentation, and ethnic density. For imputations, inpatient days and CMHS events were specified as negative binomial using “nbreg”, marital status and ethnicity were specificied as multinomial using “mlogit”, and social fragmentation and ethnic density were specified as continuous with a restricted range using “truncreg”. Variables with no missing values were included in MICE, including air pollutants, seasonality of first face-to-face contact, year of first face-to-face contact, age at first face-to-face contact, sex, population density, IMD, time in contact with SLaM, as well as additional auxiliary variables including diagnosis (F2* versus F3*) and time between first contact and diagnosis. We imputed 5 datasets using a random seed of 1234. Between N=35 (inpatient days, year 7) and N=886 (marital status) values were imputed for analysis variables.

*Population attributable fractions.* We estimated population attributable fractions (PAFs) with the use of the World Health Organization’s (WHO) formula (<https://www.who.int/quantifying_ehimpacts/faqs/en/>) for two separate exposure scenarios (South London and UK urban areas). For this purpose, we assumed causality and a log-linear exposure–response function (i.e., that relative risk remains constant per unit increase in exposure, at all concentrations).^7^ We estimated PAFs by assuming 100% exposure prevalence at a population-weighted annual mean of PM_2.5_ exposure levels for South London^8^ and UK urban (EC 2017)^9^ populations (13.4µg/m^3^ and 12.8µg/m^3^, respectively). The counterfactual scenarios we used were those of annual population-weighted average exposure being reduced to the WHO’s recommended limit for annual mean PM_2.5_ (10µg/m^3^).^10^ We selected PM_2.5_ because mean concentrations of PM_2.5_ in our study exceeded WHO’s recommended limit by a greater margin than the other air pollutants, thereby providing the clearest counterfactual scenario.


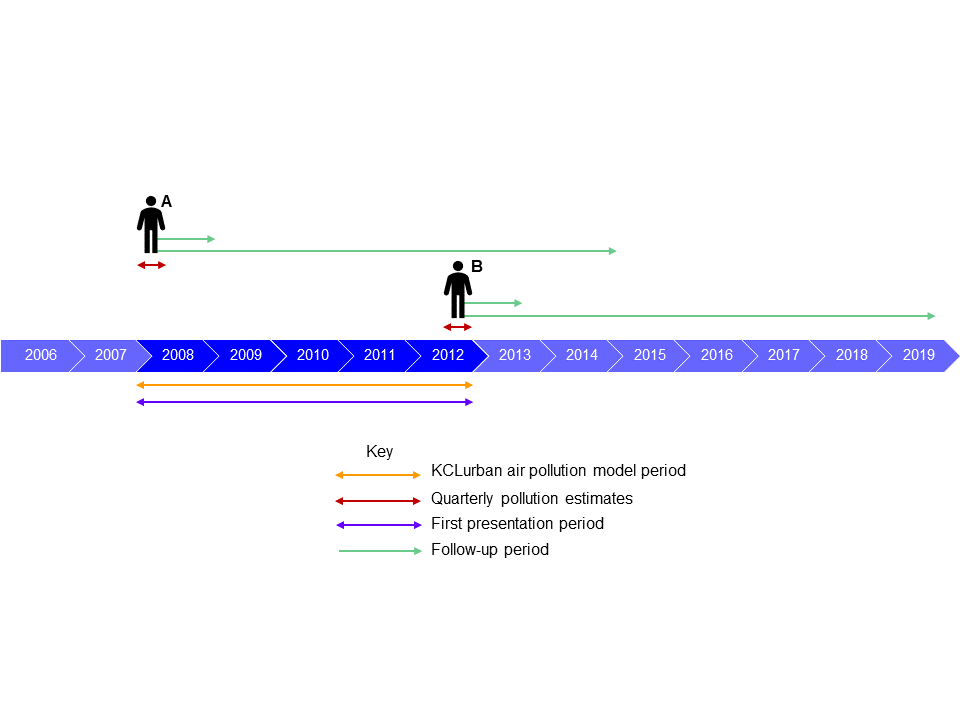


**Supplementary Figure 1.** Timeline of cohort and measures

Note: The first presentation and air pollution model periods both span 2008-2012. However, each service-user would have pollution data and a follow-up period that was unique to when they first presented to the South London and Maudsley (SLaM) NHS Foundation Trust. For instance, individual A had their first presentation at the beginning of 2008. Quarterly pollution data were linked to their address at this time, and service-use was recorded for 7 years until the start of 2015. Individual B had their first presentation at the end of 2012. Again, the quarterly pollution data were linked to their address, and service-use was recorded for 7 years until the end of 2019.


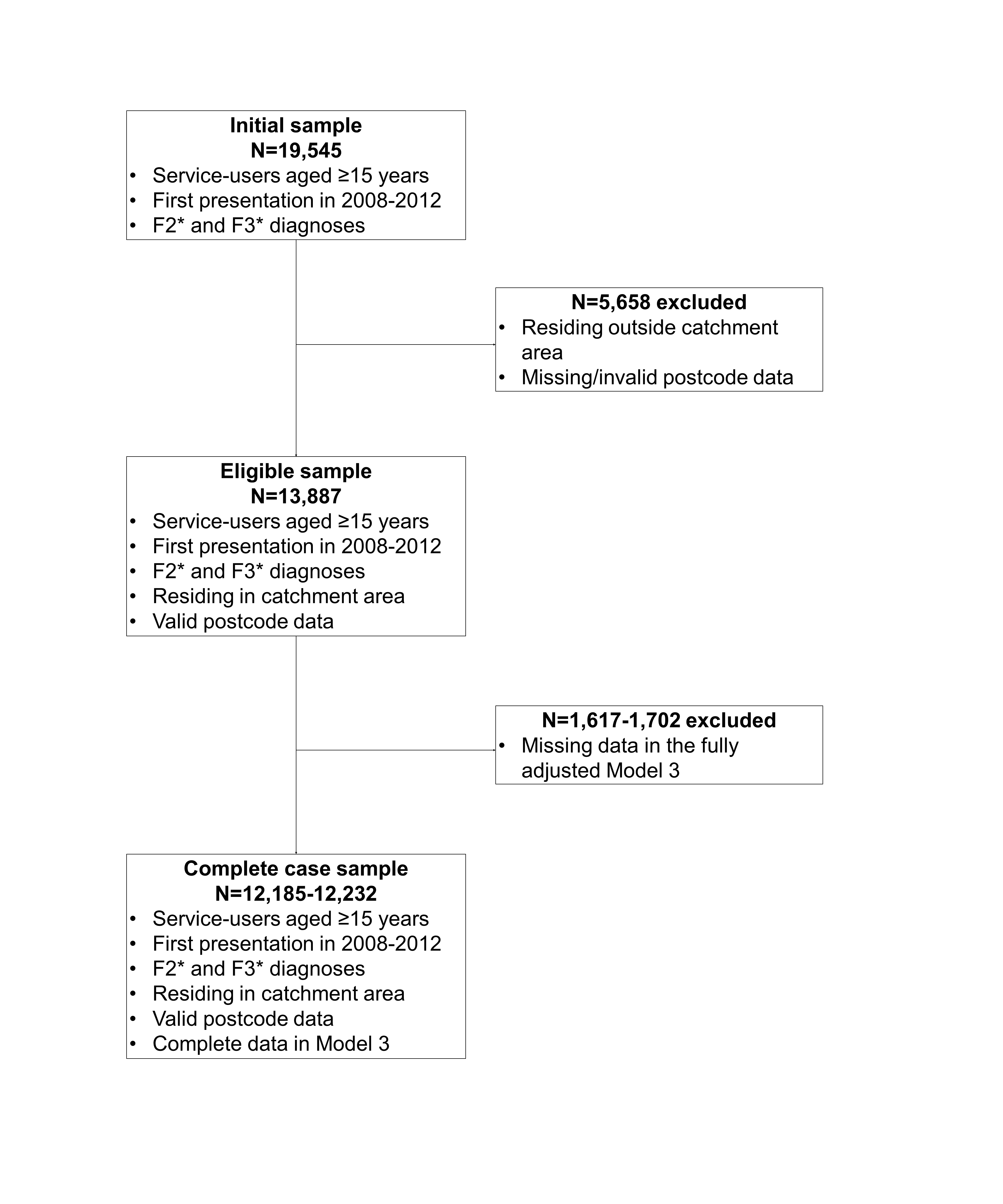


**Supplementary Figure 2.** Flow diagram of eligible and complete case sample size

**Supplementary Table 1. Association of interquartile range increases in air pollution exposure with mental health service-use over 1-year and 7-year follow-up**

| **Outcome** | **1-year follow-up** | | | | | **7-year follow-up** | | | | |
| --- | --- | --- | --- | --- | --- | --- | --- | --- | --- | --- |
| Pollutant | **N** | **Model 1** | **Model 2** | **Model 3** | | **N** | **Model 1** | **Model 2** | **Model 3** | |
|  |  | RR  (95% CI) | RR  (95% CI) | RR  (95% CI) | E-value RR  (Lower CI) |  | RR  (95% CI) | RR  (95% CI) | RR  (95% CI) | E-value RR  (Lower CI) |
| **Inpatient days** | 12,250 |  |  |  |  | 12,270 |  |  |  |  |
| NO_2_ |  | 1.28***  (1.14-1.43) | 1.24***  (1.11-1.39) | 1.18*  (1.04-1.34) | 1.64  (1.24) |  | 1.37***  (1.23-1.53) | 1.32***  (1.18-1.47) | 1.13*  (1.00-1.28) | 1.51  (1.00) |
| NO_x_ |  | 1.29***  (1.15-1.44) | 1.25***  (1.12-1.40) | 1.18**  (1.04-1.34) | 1.64  (1.24) |  | 1.32***  (1.18-1.47) | 1.27***  (1.14-1.42) | 1.09  (0.97-1.23) | 1.40  (1.00) |
| PM_2.5_ |  | 1.15***  (1.07-1.24) | 1.14***  (1.06-1.23) | 1.11**  (1.03-1.19) | 1.46  (1.21) |  | 1.22***  (1.13-1.31) | 1.18***  (1.09-1.27) | 1.10*  (1.02-1.19) | 1.43  (1.16) |
| PM_10_ |  | 1.17**  (1.06-1.29) | 1.15**  (1.04-1.27) | 1.09  (0.98-1.21) | 1.40  (1.00) |  | 1.33***  (1.20-1.48) | 1.27***  (1.14-1.41) | 1.13*  (1.01-1.25) | 1.51  (1.11) |
| **CMHS events** | 12,185 |  |  |  |  | 12,232 |  |  |  |  |
| NO_2_ |  | 1.29***  (1.24-1.35) | 1.30***  (1.24-1.35) | 1.32***  (1.25-1.38) | 1.97  (1.81) |  | 1.23***  (1.18-1.28) | 1.23***  (1.19-1.28) | 1.22***  (1.17-1.28) | 1.74  (1.62) |
| NO_x_ |  | 1.29***  (1.24-1.34) | 1.29***  (1.24-1.35) | 1.30***  (1.24-1.37) | 1.92  (1.79) |  | 1.22***  (1.18-1.27) | 1.23***  (1.18-1.27) | 1.21***  (1.16-1.26) | 1.71  (1.59) |
| PM_2.5_ |  | 1.09***  (1.06-1.12) | 1.09***  (1.06-1.12) | 1.07***  (1.04-1.11) | 1.34  (1.24) |  | 1.06***  (1.04-1.09) | 1.06***  (1.04-1.09) | 1.05***  (1.02-1.08) | 1.28  (1.16) |
| PM_10_ |  | 1.12***  (1.08-1.17) | 1.12***  (1.07-1.17) | 1.09***  (1.05-1.14) | 1.40  (1.28) |  | 1.10***  (1.06-1.14) | 1.10***  (1.06-1.14) | 1.07***  (1.03-1.11) | 1.34  (1.21) |

Note: * p<0.05; ** p<0.01; *** p<0.001; CI, confidence interval; CMHS, community mental health services; E-value, association required between unmeasured confounder(s) and both the exposure and outcome to make Model 3 effects non-significant, above and beyond measured covariates; N, complete case sample size; NO_2_, nitrogen dioxide; NO_x_, nitrogen oxides; PM_2.5_, particulate matter <2.5μm; PM_10_, particulate matter <10μm; RR, relative risk. Model 1 – adjusted for seasonality and year. Model 2 – adjusted additionally for sex, ethnicity, age and marital status. Model 3 – adjusted additionally for population density, deprivation, ethnic density, and social fragmentation. Analyses were conducted on those with complete data in Model 3.

**Supplementary Table 2. E-values versus association of covariates with inpatient days after 1-year follow-up and NO_2_**

| **Covariates** | **Association with inpatient days at 1-year follow-up** | | **Association with NO_2_** | |
| --- | --- | --- | --- | --- |
|  | **RR** | **Lower CI / 95% CI** ^a^ | **RR** | **Lower CI / 95% CI** ^a^ |
| E-value | 1.64 | 1.24 | 1.64 | 1.24 |
| Quarter |  |  |  |  |
| 1 | ref | - | ref | - |
| 2 | 1.03 | 0.92-1.16 | 0.67*** | 0.64-0.71 |
| 3 | 1.13* | 1.00-1.28 | 0.62*** | 0.58-0.65 |
| 4 | 1.48*** | 1.30-1.67 | 1.04 | 0.99-1.10 |
| Year |  |  |  |  |
| 2008 | ref | - | ref | - |
| 2009 | 0.98 | 0.86-1.12 | 1.00 | 0.94-1.07 |
| 2010 | 1.50*** | 1.31-1.72 | 1.23*** | 1.16-1.31 |
| 2011 | 0.94 | 0.83-1.07 | 1.07* | 1.01-1.14 |
| 2012 | 1.14 | 1.00-1.30 | 1.37*** | 1.28-1.47 |
| Age | 1.00*** | 1.00-100 | 1.00 | 1.00-1.00 |
| Sex |  |  |  |  |
| Female | ref | - | ref | - |
| Male | 1.17*** | 1.08-1.28 | 1.04 | 1.00-1.08 |
| Marital status |  |  |  |  |
| Married/civil partnership/cohabiting | ref | - | ref | - |
| Single | 0.99 | 0.89-1.11 | 1.06* | 1.01-1.11 |
| Divorced/civil partnership dissolved/separated | 0.97 | 0.83-1.13 | 1.03 | 0.95-1.11 |
| Widowed/surviving civil partner | 0.75* | 0.61-0.93 | 1.10* | 1.01-1.20 |
| Ethnicity |  |  |  |  |
| White British | ref | - | ref | - |
| Irish and any other White background | 1.04 | 0.90-1.20 | 0.94 | 0.88-1.01 |
| Black African | 1.34*** | 1.16-1.54 | 0.90** | 0.84-0.97 |
| Caribbean and any other Black background | 1.05 | 0.93-1.19 | 0.91** | 0.85-0.97 |
| Indian, Pakistani, and Bangladeshi | 0.86 | 0.66-1.12 | 0.79*** | 0.70-0.90 |
| White and Black Caribbean, White and Black African, White and Asian, any other mixed background, any other Asian background, Chinese, or any other ethnic group | 1.07 | 0.94-1.22 | 0.98 | 0.92-1.04 |
| Population density | 1.00 | 1.00-1.00 | 1.00*** | 1.00-1.00 |
| IMD | 0.99*** | 0.98-0.99 | 1.01*** | 1.00-1.01 |
| Ethnic density | 1.05 | 0.85-1.29 | 0.92 | 0.83-1.01 |
| Social fragmentation | 1.07*** | 1.04-1.09 | 1.05*** | 1.04-1.06 |

Note: * p<0.05; ** p<0.01; *** p<0.001; ^a^ E-values do not include p-values but the lower CI is above 1 (i.e., robust); CI, confidence interval; IMD, index of multiple deprivation; RR, relative risk; NO_2_ nitrogen dioxide.

**Supplementary Table 3. Examining specificity with psychotic versus mood disorder diagnoses**

| **Outcome** | **Association of interquartile range increases in air pollution with mental health service-use over 1-year and 7-year follow-up** | | | | | | | | | | | |
| --- | --- | --- | --- | --- | --- | --- | --- | --- | --- | --- | --- | --- |
| Pollutant | **F2* (psychotic disorder) diagnoses** | | | | | | **F3* (mood disorder) diagnoses** | | | | | |
|  | **1-year follow-up** | | | **7-year follow-up** | | | **1-year follow-up** | | | **7-year follow-up** | | |
|  | **Model 1** | **Model 2** | **Model 3** | **Model 1** | **Model 2** | **Model 3** | **Model 1** | **Model 2** | **Model 3** | **Model 1** | **Model 2** | **Model 3** |
|  | RR  (95% CI) | RR  (95% CI) | RR  (95% CI) | RR  (95% CI) | RR  (95% CI) | RR  (95% CI) | RR  (95% CI) | RR  (95% CI) | RR  (95% CI) | RR  (95% CI) | RR  (95% CI) | RR  (95% CI) |
| **Inpatient days** |  |  |  |  |  |  |  |  |  |  |  |  |
| NO_2_ | 1.22*  (1.04-1.42) | 1.16  (0.99-1.35) | 1.05  (0.89-1.25) | 1.17*  (1.00-1.37) | 1.16  (0.99-1.35) | 0.95  (0.80-1.10) | 1.26**  (1.08-1.47) | 1.25**  (1.07-1.46) | 1.26*  (1.06-1.51) | 1.46***  (1.25-1.70) | 1.41***  (1.21-1.65) | 1.27**  (1.06-1.52) |
| NO_x_ | 1.22*  (1.05-1.43) | 1.17  (1.00-1.36) | 1.06  (0.89-1.26) | 1.12  (0.96-1.31) | 1.12  (0.96-1.30) | 0.94  (0.80-1.10) | 1.27**  (1.09-1.48) | 1.27**  (1.09-1.48) | 1.27**  (1.06-1.52) | 1.41***  (1.21-1.64) | 1.37***  (1.18-1.60) | 1.22*  (1.02-1.45) |
| PM_2.5_ | 1.23***  (1.12-1.36) | 1.19***  (1.08-1.32) | 1.15**  (1.05-1.27) | 1.23***  (1.11-1.36) | 1.19**  (1.07-1.32) | 1.07  (0.97-1.18) | 1.07  (0.96-1.18) | 1.07  (0.96-1.18) | 1.04  (0.94-1.16) | 1.15**  (1.04-1.28) | 1.13*  (1.02-1.26) | 1.08  (0.97-1.20) |
| PM_10_ | 1.25**  (1.08-1.43) | 1.19**  (1.03-1.36) | 1.12  (0.97-1.29) | 1.28***  (1.11-1.48) | 1.24**  (1.07-1.43) | 1.07  (0.93-1.22) | 1.06  (0.92-1.23) | 1.06  (0.92-1.23) | 1.02  (0.88-1.19) | 1.27**  (1.09-1.47) | 1.22**  (1.05-1.42) | 1.12  (0.96-1.30) |
| **CMHS events** |  |  |  |  |  |  |  |  |  |  |  |  |
| NO_2_ | 1.34***  (1.23-1.44) | 1.32***  (1.22-1.43) | 1.23***  (1.12-1.34) | 1.20***  (1.12-1.28) | 1.21***  (1.14-1.29) | 1.16***  (1.08-1.25) | 1.26***  (1.20-1.32) | 1.29***  (1.22-1.36) | 1.35***  (1.28-1.43) | 1.26***  (1.20-1.32) | 1.29***  (1.23-1.36) | 1.35***  (1.28-1.43) |
| NO_x_ | 1.32***  (1.22-1.43) | 1.31***  (1.21-1.41) | 1.21***  (1.11-1.32) | 1.19***  (1.11-1.27) | 1.20***  (1.13-1.28) | 1.15***  (1.07-1.24) | 1.26***  (1.20-1.32) | 1.29***  (1.23-1.35) | 1.34***  (1.26-1.42) | 1.26***  (1.20-1.32) | 1.29***  (1.23-1.35) | 1.34***  (1.26-1.42) |
| PM_2.5_ | 1.13***  (1.07-1.19) | 1.12***  (1.06-1.19) | 1.09**  (1.03-1.15) | 1.08**  (1.03-1.13) | 1.08**  (1.03-1.13) | 1.06*  (1.02-1.11) | 1.07***  (1.03-1.11) | 1.07***  (1.04-1.11) | 1.07***  (1.03-1.11) | 1.07***  (1.03-1.11) | 1.07***  (1.04-1.11) | 1.07**  (1.03-1.10) |
| PM_10_ | 1.19***  (1.10-1.28) | 1.18***  (1.09-1.27) | 1.11**  (1.03-1.20) | 1.10**  (1.03-1.18) | 1.11**  (1.04-1.18) | 1.07  (1.01-1.15) | 1.09***  (1.04-1.14) | 1.10***  (1.05-1.15) | 1.08**  (1.03-1.14) | 1.09***  (1.04-1.14) | 1.10***  (1.05-1.15) | 1.08**  (1.03-1.14) |

Note: * p<0.05; ** p<0.01; *** p<0.001; CI, confidence interval; CMHS, community mental health services; N, complete case sample size; NO_2_, nitrogen dioxide; NO_x_, nitrogen oxides; PM_2.5_, particulate matter <2.5μm; PM_10_, particulate matter <10μm; RR, relative risk. Model 1 – adjusted only for seasonality and year. Model 2 – adjusted additionally for sex, ethnicity, age and marital status. Model 3 – adjusted additionally for population density, deprivation, ethnic density, and social fragmentation. Analyses were conducted on those with complete data in Model 3. Within F2* diagnoses, this sample size was N=2,890 for inpatient days, Year 1; N=2,896 for inpatient days, Year 7; N=2,867 for CMHS events, Year 1; and N=2,886 for CMHS events, Year 7. Within F3* diagnoses, this sample size was N=9,360 for inpatient days, Year 1; N=9,374 for inpatient days, Year 7; N=9,318 for CMHS events, Year 1; and N=9,346 for CMHS events, Year 7.

**Supplementary Table 4. Examining the impact of missing data by conducting multiple imputation by chained equations**

| **Outcome** | **Association of interquartile range increases in air pollution exposure with mental health service-use over 1-year and 7-year follow-up** | | | | | | | |
| --- | --- | --- | --- | --- | --- | --- | --- | --- |
| Pollutant | **1-year follow-up** | | | | **7-year follow-up** | | | |
|  | **N** | **Model 1** | **Model 2** | **Model 3** | **N** | **Model 1** | **Model 2** | **Model 3** |
|  |  | RR  (95% CI) | RR  (95% CI) | RR  (95% CI) |  | RR  (95% CI) | RR  (95% CI) | RR  (95% CI) |
| **Inpatient days** | 13,887 |  |  |  | 13,887 |  |  |  |
| NO_2_ |  | 1.27***  (1.13-1.42) | 1.22***  (1.09-1.31) | 1.16*  (1.03-1.31) |  | 1.32***  (1.19-1.47) | 1.26***  (1.14-1.41) | 1.11  (0.98-1.26) |
| NO_x_ |  | 1.27***  (1.13-1.42) | 1.22***  (1.09-1.36) | 1.15*  (1.03-1.30) |  | 1.26***  (1.14-1.41) | 1.21***  (1.09-1.35) | 1.06  (0.94-1.20) |
| PM_2.5_ |  | 1.13**  (1.05-1.21) | 1.11**  (1.03-1.19) | 1.08*  (1.01-1.16) |  | 1.16***  (1.07-1.25) | 1.13**  (1.04-1.21) | 1.06  (0.98-1.14) |
| PM_10_ |  | 1.15*  (1.03-1.29) | 1.12*  (1.00-1.25) | 1.07  (0.96-1.20) |  | 1.26*  (1.14-1.40) | 1.20*  (1.09-1.34) | 1.08  (0.98-1.21) |
| **CMHS events** | 13,887 |  |  |  | 13,887 |  |  |  |
| NO_2_ |  | 1.24***  (1.19-1.29) | 1.25***  (1.20-1.30) | 1.26***  (1.20-1.32) |  | 1.18***  (1.14-1.23) | 1.19***  (1.15-1.24) | 1.17***  (1.12-1.23) |
| NO_x_ |  | 1.24***  (1.19-1.28) | 1.24***  (1.20-1.29) | 1.25***  (1.19-1.31) |  | 1.17***  (1.13-1.22) | 1.18***  (1.14-1.23) | 1.16***  (1.11-1.21) |
| PM_2.5_ |  | 1.08***  (1.05-1.11) | 1.08***  (1.05-1.11) | 1.07***  (1.04-1.10) |  | 1.04**  (1.02-1.07) | 1.05***  (1.02-1.07) | 1.03*  (1.00-1.06) |
| PM_10_ |  | 1.11***  (1.06-1.15) | 1.11***  (1.06-1.15) | 1.09***  (1.06-1.15) |  | 1.07***  (1.04-1.11) | 1.08***  (1.04-1.12) | 1.05*  (1.01-1.09) |

Note: * p<0.05; ** p<0.01; *** p<0.001; CI, confidence interval; CMHS, community mental health services; N, complete case sample size; NO_2_, nitrogen dioxide; NO_x_, nitrogen oxides; PM_2.5_, particulate matter <2.5μm; PM_10_, particulate matter <10μm; RR, relative risk. Model 1 – adjusted only for seasonality and year. Model 2 – adjusted additionally for sex, ethnicity, age and marital status. Model 3 – adjusted additionally for population density, deprivation, ethnic density, and social fragmentation. Analyses were conducted following multiple imputation by chained equations (MICE).

**Supplementary Table 5. Examining interactions between neighbourhood deprivation and air pollution**

| **Outcome** | **Association of interquartile range increases in air pollution exposure with mental health service-use over 1-year and 7-year follow-up** | | | | | | | | | |
| --- | --- | --- | --- | --- | --- | --- | --- | --- | --- | --- |
| Pollutant | **1-year follow-up** | | | | | **7-year follow-up** | | | | |
|  | **N** | **Interactions across IMD quartiles^a^** | | | | **N** | **Interactions across IMD quartiles^a^** | | | |
|  |  | **Q1 (lowest)** | **Q2** | **Q3** | **Q4 (highest)** |  | **Q1 (lowest)** | **Q2** | **Q3** | **Q4 (highest)** |
|  |  | (ref) | RR  (95% CI) | RR  (95% CI) | RR  (95% CI) |  | (ref) | RR  (95% CI) | RR  (95% CI) | RR  (95% CI) |
| **Inpatient days** | 12,250 |  |  |  |  | 12,270 |  |  |  |  |
| NO_2_ |  | - | 1.01  (0.83-1.23) | 0.88  (0.73-1.07) | 0.88  (0.72-1.06) |  | - | 0.88  (0.72-1.07) | 0.98  (0.81-1.20) | 0.81*  (0.66-0.99) |
| NO_x_ |  | - | 1.03  (0.84-1.25) | 0.88  (0.73-1.07) | 0.88  (0.72-1.07) |  | - | 0.91  (0.74-1.11) | 1.01  (0.83-1.24) | 0.82  (0.67-1.01) |
| PM_2.5_ |  | - | 1.01  (0.91-1.13) | 0.98  (0.89-1.09) | 1.02  (0.91-1.14) |  | - | 1.02  (0.91-1.13) | 0.94  (0.84-1.04) | 0.97  (0.86-1.08) |
| PM_10_ |  | - | 0.93  (0.80-1.09) | 0.97  (0.83-1.13) | 1.05  (0.89-1.23) |  | - | 0.93  (0.79-1.08) | 0.86  (0.74-1.01) | 0.91  (0.78-1.08) |
| **CMHS events** | 12,185 |  |  |  |  | 12,232 |  |  |  |  |
| NO_2_ |  | - | 0.88**  (0.81-0.95) | 0.92*  (0.85-0.99) | 0.84***  (0.78-0.91) |  | - | 0.94  (0.88-1.01) | 0.93*  (0.86-0.99) | 0.92*  (0.85-0.98) |
| NO_x_ |  | - | 0.87***  (0.80-0.94) | 0.90**  (0.83-0.97) | 0.83***  (0.77-0.90) |  | - | 0.93  (0.87-1.00) | 0.91**  (0.85-0.97) | 0.90**  (0.84-0.97) |
| PM_2.5_ |  | - | 1.02  (0.98-1.07) | 1.03  (0.98-1.07) | 0.97  (0.93-1.02) |  | - | 1.02  (0.98-1.06) | 1.01  (0.97-1.05) | 0.99  (0.95-1.03) |
| PM_10_ |  | - | 1.02  (0.95-1.08) | 1.02  (0.95-1.08) | 0.98  (0.92-1.05) |  | - | 1.05  (0.99-1.11) | 1.00  (0.94-1.06) | 1.03  (0.97-1.09) |

Note: * p<0.05; ** p<0.01; *** p<0.001; ^a^the interaction coefficients represent the value by which the main effects should be multiplied within each deprivation quartile, using least deprived as the reference; CI, confidence interval; CMHS, community mental health services; IMD, index of multiple deprivation; N, complete case sample size; NO_2_, nitrogen dioxide; NO_x_, nitrogen oxides; PM_2.5_, particulate matter <2.5μm; PM_10_, particulate matter <10μm; RR, relative risk; Q1-Q4 quartiles of deprivation according to IMD. All models are adjusted for seasonality, year, sex, ethnicity, age, marital status, population density, ethnic density, and social fragmentation. Analyses were conducted on those with complete covariate data.

**Supplementary Table 6. Examining interactions between borough of residence and air pollution**

| **Outcome** | **Association of interquartile range increases in air pollution exposure with mental health service-use over 1-year and 7-year follow-up** | | | | | | | | | | |
| --- | --- | --- | --- | --- | --- | --- | --- | --- | --- | --- | --- |
| Pollutant | **1-year follow-up** | | | | | **7-year follow-up** | | | | | |
|  | **N** | **Interactions across boroughs^a^** | | | | **N** | **Interactions across boroughs^a^** | | | | |
|  |  | **Southwark** | **Lambeth** | **Lewisham** | **Croydon** |  | **Southwark** | **Lambeth** | **Lewisham** | **Croydon** |  |
|  |  | (ref) | RR  (95% CI) | RR  (95% CI) | RR  (95% CI) |  | (ref) | RR  (95% CI) | RR  (95% CI) | RR  (95% CI) |  |
| **Inpatient days** | 12,250 |  |  |  |  | 12,270 |  |  |  |  |  |
| NO_2_ |  | - | 0.93  (0.77-1.12) | 1.08  (0.89-1.31) | 1.42**  (1.17-1.73) |  | - | 0.90  (0.74-1.09) | 1.05  (0.86-1.27) | 1.12  (0.92-1.36) |  |
| NO_x_ |  | - | 0.94  (0.78-1.12) | 1.09  (0.90-1.31) | 1.51***  (1.23-1.85) |  | - | 0.91  (0.75-1.09) | 1.04  (0.85-1.26) | 1.11  (0.90-1.37) |  |
| PM_2.5_ |  | - | 0.96  (0.86-1.06) | 1.02  (0.92-1.14) | 0.98  (0.88-1.09) |  | - | 1.00  (0.90-1.12) | 1.04  (0.93-1.16) | 0.99  (0.89-1.10) |  |
| PM_10_ |  | - | 0.95  (0.82-1.11) | 1.09  (0.73-0.99) | 0.85*  (0.73-0.99) |  | - | 1.07  (0.91-1.24) | 1.07  (0.91-1.25) | 0.87  (0.75-1.01) |  |
| **CMHS events** | 12,185 |  |  |  |  | 12,232 |  |  |  |  |  |
| NO_2_ |  | - | 0.99  (0.92-1.06) | 0.91*  (0.84-0.99) | 0.98  (0.90-1.06) |  | - | 0.98  (0.92-1.05) | 0.93*  (0.86-0.99) | 0.95  (0.88-1.02) |  |
| NO_x_ |  | - | 0.99  (0.92-1.06) | 0.93  (0.85-1.00) | 1.01  (0.93-1.10) |  | - | 0.98  (0.92-1.04) | 0.94  (0.87-1.00) | 0.96  (0.89-1.03) |  |
| PM_2.5_ |  | - | 0.98  (0.94-1.02) | 0.96  (0.91-1.00) | 0.94**  (0.89-0.98) |  | - | 1.00  (0.96-1.04) | 0.99  (0.95-1.03) | 0.96  (0.93-1.00) |  |
| PM_10_ |  | - | 0.99  (0.94-1.03) | 0.98  (0.92-1.04) | 1.21***  (1.12-1.31) |  | - | 1.02  (0.97-1.08) | 0.96  (0.91-1.02) | 0.92**  0.87-0.98) |  |

Note: * p<0.05; ** p<0.01; *** p<0.001; ^a^the interaction coefficients represent the value by which the main effects should be multiplied within each borough, using Southwark as the reference; CI, confidence interval; CMHS, community mental health services; IMD, index of multiple deprivation; N, complete case sample size; NO_2_, nitrogen dioxide; NO_x_, nitrogen oxides; PM_2.5_, particulate matter <2.5μm; PM_10_, particulate matter <10μm; RR, relative risk; All models are adjusted for seasonality, year, sex, ethnicity, age, marital status, population density, deprivation, ethnic density, and social fragmentation. Analyses were conducted on those with complete covariate data.

**Supplementary Table 7. Two-pollutant model**

| **Outcome** | **Association of interquartile range increases in air pollution exposure with mental health service-use over 1-year and 7-year follow-up** | | | | | | | | | | |
| --- | --- | --- | --- | --- | --- | --- | --- | --- | --- | --- | --- |
| Pollutant | **1-year follow-up** | | | | | **7-year follow-up** | | | | | |
|  | **N** | ***Co-pollutant confounder added to Model 3*** | | | | **N** | ***Co-pollutant confounder added to Model 3*** | | | | |
|  |  | ***NO_2_*** | ***NO_x_*** | ***PM_2.5_*** | ***PM_10_*** |  | ***NO_2_*** | ***NO_x_*** | ***PM_2.5_*** | ***PM_10_*** |  |
|  |  | RR  (95% CI) | RR  (95% CI) | RR  (95% CI) | RR  (95% CI) |  | RR  (95% CI) | RR  (95% CI) | RR  (95% CI) | RR  (95% CI) |  |
| **Inpatient days** | 12,250 |  |  |  |  | 12,270 |  |  |  |  |  |
| NO_2_ |  | - | 0.91  (0.45-1.86) | 1.09  (0.93-1.28) | 1.20  (1.00-1.43) |  | - | 3.39***  (1.70-6.76) | 1.04  (0.89-1.21) | 1.04  (0.88-1.24) |  |
| NO_x_ |  | 1.29  (0.64-2.64) | - | 1.10  (0.94-1.29) | 1.20*  (1.01-1.42) |  | 0.33**  (0.17-0.65) | - | 0.98  (0.85-1.14) | 0.99  (0.84-1.16) |  |
| PM_2.5_ |  | 1.07  (0.98-1.18) | 1.07  (0.98-1.17) | - | 1.41***  (1.16-1.71) |  | 1.09  (0.99-1.19) | 1.10*  (1.01-1.21) | - | 1.13  (0.94-1.37) |  |
| PM_10_ |  | 0.98  (0.84-1.13) | 0.98  (0.86-1.13) | 0.69**  (0.52-0.91) | - |  | 1.10  (0.95-1.27) | 1.13  (0.95-1.27) | 0.96  (0.73-1.25) | - |  |
| **CMHS events** | 12,185 |  |  |  |  | 12,232 |  |  |  |  |  |
| NO_2_ |  | - | 1.33*  (1.01-1.76) | 1.41***  (1.32-1.51) | 1.51***  (1.41-1.62) |  | - | 1.40**  (1.10-1.78) | 1.30***  (1.23-1.38) | 1.36***  (1.27-1.44) |  |
| NO_x_ |  | 0.99  (0.75-1.29) | - | 1.38***  (1.29-1.46) | 1.44***  (1.29-1.46) |  | 0.88  (0.69-1.11) | - | 1.27***  (1.20-1.34) | 1.30***  (1.22-1.37) |  |
| PM_2.5_ |  | 0.94***  (0.90-0.97) | 0.95**  (0.91-0.98) | - | 1.08*  (1.00-1.17) |  | 0.95**  (0.91-0.98) | 0.96*  (0.93-0.99) | - | 1.02  (0.95-1.09) |  |
| PM_10_ |  | 0.84***  (0.79-0.90) | 0.88***  (0.83-0.93) | 0.98  (0.88-1.10) | - |  | 0.89***  (0.84-0.94) | 0.92***  (0.87-0.97) | 1.04  (0.95-1.15) | - |  |

Note: * p<0.05; ** p<0.01; *** p<0.001; CI, confidence interval; CMHS, community mental health services; N, complete case sample size; NO_2_, nitrogen dioxide; NO_x_, nitrogen oxides; PM_2.5_, particulate matter <2.5μm; PM_10_, particulate matter <10μm; RR, relative risk. All models are adjusted for seasonality, year, sex, ethnicity, age, marital status, population density, deprivation, ethnic density, and social fragmentation, in addition to co-pollutant confounders. Analyses were conducted on those with complete covariate data.

**Supplementary Table 8. Examining diagnosis uncertainty**

| **Outcome** | **Association of interquartile range increases in air pollution with mental health service-use over 1-year and 7-year follow-up** | | | | | | | |
| --- | --- | --- | --- | --- | --- | --- | --- | --- |
| Pollutant | **1-year follow-up** | | | | **7-year follow-up** | | | |
|  | **N** | **Model 1** | **Model 2** | **Model 3** | **N** | **Model 1** | **Model 2** | **Model 3** |
|  |  | RR  (95% CI) | RR  (95% CI) | RR  (95% CI) |  | RR  (95% CI) | RR  (95% CI) | RR  (95% CI) |
| **Inpatient days** | 5,684 |  |  |  | 5,687 |  |  |  |
| NO_2_ |  | 1.72***  (1.43-2.07) | 1.48***  (1.23-1.78) | 1.31**  (1.07-1.62) |  | 1.90***  (1.57-2.29) | 1.61**  (1.33-1.95) | 1.28*  (1.04-1.57) |
| NO_x_ |  | 1.78***  (1.48-2.14) | 1.52***  (1.26-1.82) | 1.35**  (1.10-1.66) |  | 1.82***  (1.51-2.19) | 1.55***  (1.29-1.87) | 1.24*  (1.01-1.52) |
| PM_2.5_ |  | 1.31***  (1.17-1.48) | 1.22***  (1.09-1.38) | 1.17**  (1.04-1.31) |  | 1.52***  (1.34-1.72) | 1.40***  (1.23-1.59) | 1.25***  (1.10-1.41) |
| PM_10_ |  | 1.42***  (1.20-1.69) | 1.29**  (1.09-1.53) | 1.17  (0.99-1.39) |  | 1.86***  (1.56-2.22) | 1.64***  (1.38-1.96) | 1.34**  (1.12-1.61) |
| **CMHS events** | 5,646 |  |  |  | 5,657 |  |  |  |
| NO_2_ |  | 1.44***  (1.35-1.53) | 1.43***  (1.35-1.53) | 1.40***  (1.29-1.50) |  | 1.34***  (1.27-1.43) | 1.36***  (1.28-1.44) | 1.31***  (1.22-1.40) |
| NO_x_ |  | 1.44***  (1.35-1.54) | 1.44***  (1.35-1.54) | 1.39***  (1.29-1.50) |  | 1.34***  (1.26-1.42) | 1.35***  (1.27-1.43) | 1.29***  (1.21-1.39) |
| PM_2.5_ |  | 1.17***  (1.11-1.22) | 1.16***  (1.11-1.22) | 1.13***  (1.08-1.18) |  | 1.14***  (1.10-1.19) | 1.14***  (1.10-1.19) | 1.11***  (1.07-1.16) |
| PM_10_ |  | 1.23***  (1.15-1.31) | 1.22***  (1.15-1.30) | 1.16***  (1.08-1.24) |  | 1.21***  (1.14-1.29) | 1.21***  (1.14-1.28) | 1.16***  (1.09-1.23) |

Note: * p<0.05; ** p<0.01; *** p<0.001; CI, confidence interval; CMHS, community mental health services; N, complete case sample size; NO_2_, nitrogen dioxide; NO_x_, nitrogen oxides; PM_2.5_, particulate matter <2.5μm; PM_10_, particulate matter <10μm; RR, relative risk. Model 1 – adjusted only for seasonality and year. Model 2 – adjusted additionally for sex, ethnicity, age and marital status. Model 3 – adjusted additionally for population density, deprivation, ethnic density, and social fragmentation. Analyses were conducted on those with complete data in Model 3 who were diagnosed within 30 days of first face-to-face contact in SLaM.

**Supplementary Table 9. Examining the impact of residential mobility by restricting to service-users who had not moved house after 3 months following first contact with SLaM**

| **Outcome** | **1-year follow-up** | | | | **7-year follow-up** | | | |
| --- | --- | --- | --- | --- | --- | --- | --- | --- |
| Pollutant | **N** | **Model 1** | **Model 2** | **Model 3** | **N** | **Model 1** | **Model 2** | **Model 3** |
|  |  | RR  (95% CI) | RR  (95% CI) | RR  (95% CI) |  | RR  (95% CI) | RR  (95% CI) | RR  (95% CI) |
| **Inpatient days** | 10,339 |  |  |  | 10,353 |  |  |  |
| NO_2_ |  | 1.25***  (1.11-1.41) | 1.27***  (1.12-1.42) | 1.16*  (1.01-1.32) |  | 1.32***  (1.17-1.48) | 1.31***  (1.16-1.47) | 1.15*  (1.01-1.32) |
| NO_x_ |  | 1.26***  (1.12-1.41) | 1.26***  (1.12-1.43) | 1.16*  (1.01-1.32) |  | 1.30***  (1.16-1.46) | 1.29***  (1.14-1.45) | 1.13  (0.99-1.29) |
| PM_2.5_ |  | 1.05  (0.97-1.14) | 1.06  (0.97-1.14) | 1.03  (0.95-1.12) |  | 1.08  (0.99-1.17) | 1.08  (0.99-1.17) | 1.05  (0.96-1.13) |
| PM_10_ |  | 1.05  (0.94-1.18) | 1.06  (0.95-1.19) | 1.01  (0.90-1.13) |  | 1.12*  (1.01-1.26) | 1.11  (1.00-1.25) | 1.05  (0.93-1.17) |
| **CMHS events** | 10,281 |  |  |  | 10,324 |  |  |  |
| NO_2_ |  | 1.29***  (1.23-1.35) | 1.30***  (1.24-1.36) | 1.32***  (1.25-1.39) |  | 1.24***  (1.19-1.29) | 1.25***  (1.20-1.30) | 1.24***  (1.19-1.30) |
| NO_x_ |  | 1.29***  (1.23-1.34) | 1.29***  (1.24-1.35) | 1.30***  (1.24-1.37) |  | 1.23***  (1.18-1.28) | 1.24***  (1.19-1.29) | 1.23***  (1.17-1.29) |
| PM_2.5_ |  | 1.07***  (1.03-1.10) | 1.07***  (1.03-1.10) | 1.05**  (1.02-1.09) |  | 1.06***  (1.03-1.09) | 1.06***  (1.03-1.09) | 1.04**  (1.01-1.07) |
| PM_10_ |  | 1.10***  (1.05-1.15) | 1.10***  (1.05-1.15) | 1.08**  (1.03-1.13) |  | 1.09***  (1.05-1.14) | 1.10***  (1.05-1.14) | 1.07**  (1.03-1.11) |

Note: * p<0.05; ** p<0.01; *** p<0.001; CI, confidence interval; CMHS, community mental health services; E-value, association required between unmeasured confounder(s) and both the exposure and outcome to make Model 3 effects non-significant, above and beyond measured covariates; N, complete case sample size; NO_2_, nitrogen dioxide; NO_x_, nitrogen oxides; PM_2.5_, particulate matter <2.5μm; PM_10_, particulate matter <10μm; RR, relative risk. Model 1 – adjusted for seasonality and year. Model 2 – adjusted additionally for sex, ethnicity, age and marital status. Model 3 – adjusted additionally for population density, deprivation, ethnic density, and social fragmentation. Analyses were conducted on those with complete data in Model 3.

**Supplementary Table 10. Examining the impact of residential mobility by using annualised data and restricting to those who had not moved house after 1 year following first contact with SLaM**

| **Outcome** | **1-year follow-up** | | | | **7-year follow-up** | | | |
| --- | --- | --- | --- | --- | --- | --- | --- | --- |
| Pollutant | **N** | **Model 1** | **Model 2** | **Model 3** | **N** | **Model 1** | **Model 2** | **Model 3** |
|  |  | RR  (95% CI) | RR  (95% CI) | RR  (95% CI) |  | RR  (95% CI) | RR  (95% CI) | RR  (95% CI) |
| **Inpatient days** | 8,595 |  |  |  | 8,609 |  |  |  |
| NO_2_ |  | 1.21***  (1.12-1.31) | 1.21***  (1.12-1.31) | 1.13*  (1.03-1.25) |  | 1.32***  (1.22-1.42) | 1.32***  (1.21-1.41) | 1.22***  (1.11-1.34) |
| NO_x_ |  | 1.22***  (1.12-1.32) | 1.22***  (1.12-1.32) | 1.13*  (1.02-1.25) |  | 1.32***  (1.22-1.42) | 1.30***  (1.20-1.41) | 1.21***  (1.09-1.33) |
| PM_2.5_ |  | 1.44***  (0.21-1.72) | 1.45***  (1.21-1.73) | 1.21  (0.98-1.51) |  | 1.71***  (1.45-2.02) | 1.66***  (1.40-1.97) | 1.36**  (1.11-1.68) |
| PM_10_ |  | 1.39***  (1.19-1.61) | 1.39***  (1.19-1.62) | 1.20*  (1.01-1.44) |  | 1.64***  (1.41-1.89) | 1.59***  (1.37-1.84) | 1.36***  (1.15-1.63) |
| **CMHS events** | 8,540 |  |  |  | 8,580 |  |  |  |
| NO_2_ |  | 1.16***  (1.12-1.19) | 1.17***  (1.14-1.20) | 1.23***  (1.18-1.27) |  | 1.13***  (1.10-1.16) | 1.14***  (1.11-1.17) | 1.16***  (1.13-1.20) |
| NO_x_ |  | 1.16***  (1.13-1.19) | 1.17***  (1.14-1.21) | 1.23***  (1.18-1.27) |  | 1.13***  (1.10-1.16) | 1.14***  (1.11-1.17) | 1.16***  (1.12-1.20) |
| PM_2.5_ |  | 1.33**  (1.25-1.41) | 1.36***  (1.27-1.44) | 1.45***  (1.34-1.57) |  | 1.27***  (1.20-1.34) | 1.30***  (1.23-1.37) | 1.31***  (1.22-1.41) |
| PM_10_ |  | 1.29***  (1.22-1.36) | 1.31***  (1.25-1.39) | 1.39***  (1.29-1.48) |  | 1.24***  (1.18-1.30) | 1.26***  (1.20-1.33) | 1.27***  (1.20-1.35) |

Note: * p<0.05; ** p<0.01; *** p<0.001; CI, confidence interval; CMHS, community mental health services; E-value, association required between unmeasured confounder(s) and both the exposure and outcome to make Model 3 effects non-significant, above and beyond measured covariates; N, complete case sample size; NO_2_, nitrogen dioxide; NO_x_, nitrogen oxides; PM_2.5_, particulate matter <2.5μm; PM_10_, particulate matter <10μm; RR, relative risk. Model 1 – adjusted for seasonality and year. Model 2 – adjusted additionally for sex, ethnicity, age and marital status. Model 3 – adjusted additionally for population density, deprivation, ethnic density, and social fragmentation. Analyses were conducted on those with complete data in Model 3.

**Supplementary Table 11. Examining the impact of residential mobility by using annualised data and restricting to those who had not moved house after 7 years following first contact with SLaM**

| **Outcome** | **1-year follow-up** | | | | **7-year follow-up** | | | |
| --- | --- | --- | --- | --- | --- | --- | --- | --- |
| Pollutant | **N** | **Model 1** | **Model 2** | **Model 3** | **N** | **Model 1** | **Model 2** | **Model 3** |
|  |  | RR  (95% CI) | RR  (95% CI) | RR  (95% CI) |  | RR  (95% CI) | RR  (95% CI) | RR  (95% CI) |
| **Inpatient days** | 4,606 |  |  |  | 4,606 |  |  |  |
| NO_2_ |  | 1.31***  (1.17-1.46) | 1.24***  (1.11-1.40) | 1.21**  (1.05-1.39) |  | 1.44***  (1.26-1.64) | 1.30***  (1.14-1.49) | 1.22*  (1.03-1.44) |
| NO_x_ |  | 1.31***  (1.17-1.47) | 1.24***  (1.10-1.40) | 1.20*  (1.03-1.39) |  | 1.44***  (1.25-1.65) | 1.29***  (1.13-1.48) | 1.20*  (1.01-1.42) |
| PM_2.5_ |  | 1.69***  (1.31-2.19) | 1.51***  (1.17-1.96) | 1.35  (0.99-1.85) |  | 2.07***  (1.53-2.79) | 1.64**  (1.21-2.22) | 1.35  (0.94-1.95) |
| PM_10_ |  | 1.62***  (1.30-2.01) | 1.47***  (1.18-1.83) | 1.35*  (1.04-1.75) |  | 1.93***  (1.49-2.49) | 1.58***  (1.22-2.05) | 1.36*  (1.01-1.85) |
| **CMHS events** | 4,572 |  |  |  | 4,577 |  |  |  |
| NO_2_ |  | 1.13***  (1.09-1.18) | 1.16***  (1.12-1.21) | 1.24***  (1.18-1.30) |  | 1.12***  (1.08-1.16) | 1.15***  (1.10-1.19) | 1.19***  (1.13-1.25) |
| NO_x_ |  | 1.14***  (1.09-1.18) | 1.17***  (1.12-1.21) | 1.24***  (1.17-1.30) |  | 1.12***  (1.08-1.16) | 1.15***  (1.10-1.19) | 1.19***  (1.13-1.25) |
| PM_2.5_ |  | 1.27***  (1.17-1.39) | 1.34***  (1.23-1.47) | 1.48***  (1.32-1.65) |  | 1.23***  (1.13-1.34) | 1.30***  (1.19-1.41) | 1.35***  (1.21-1.51) |
| PM_10_ |  | 1.25***  (1.16-1.34) | 1.31***  (1.21-1.41) | 1.41***  (1.28-1.55) |  | 1.21***  (1.13-1.30) | 1.27***  (1.18-1.36) | 1.31***  (1.19-1.44) |

Note: * p<0.05; ** p<0.01; *** p<0.001; CI, confidence interval; CMHS, community mental health services; E-value, association required between unmeasured confounder(s) and both the exposure and outcome to make Model 3 effects non-significant, above and beyond measured covariates; N, complete case sample size; NO_2_, nitrogen dioxide; NO_x_, nitrogen oxides; PM_2.5_, particulate matter <2.5μm; PM_10_, particulate matter <10μm; RR, relative risk. Model 1 – adjusted for seasonality and year. Model 2 – adjusted additionally for sex, ethnicity, age and marital status. Model 3 – adjusted additionally for population density, deprivation, ethnic density, and social fragmentation. Analyses were conducted on those with complete data in Model 3.

**Supplementary Table 12. Population attributable fractions (PAF) and relative risk calculations associated with current versus counterfactual PM_2.5_ exposure**

| **Association of PM_2.5_ with mental health service-use** | | **Scenario: South London** | | | | **Scenario: UK urban areas** | | | |
| --- | --- | --- | --- | --- | --- | --- | --- | --- | --- |
|  |  | Mean population weighted exposure (µg/m^3^) in 2019 | | | | Mean population weighted exposure (µg/m^3^) in 2017 | | | |
|  | RR  (95% CI) | Current^a^ | Counterfactual^b^ | RR  (95% CI)^c^ | PAF (%)^d^  (95% CI) | Current^a^ | Counterfactual^b^ | RR (95% CI)^c^ | PAF (%)^d^  (95% CI) |
| Inpatient days | 1.11  (1.03-1.19) | 13.4 | 10.0 | 1.03  (1.01-1.06) | 2.9  (0.9-5.8) | 12.8 | 10 | 1.02  (1.01-1.04) | 2.0  (0.9-3.8) |
| CMHS events | 1.07  (1.04-1.11) | 13.4 | 10.0 | 1.02  (1.01-1.03) | 2.0  (1.0-2.9) | 12.8 | 10 | 1.02  (1.01-1.03) | 1.9  (0.9-2.9) |

Note: ^a^ Current average concentrations; ^b^ ­­World Health Organization’s annual PM_2.5_ exposure threshold; ^c^ ­­Current vs counterfactual; ^d^ (RR-1)/RR (95% CI); CI, confidence interval; CMHS, community mental health services; PAF, population attributable fraction; RR, relative risk

**References from Supplementary Materials**

1. Office of National Statistics. Census geography: Office of National Statistics; 2016 [Available from: <https://www.ons.gov.uk/methodology/geography/ukgeographies/censusgeography>.]

2. Office of National Statistics. 2011 Census: Population and Household Estimates for England and Wales: Office of National Statistics; 2011 [Available from: <https://www.ons.gov.uk/peoplepopulationandcommunity/populationandmigration/populationestimates/datasets/2011censuspopulationandhouseholdestimatesforenglandandwales>.]

3. Department for Communities and Local Government. *The English Indices of Deprivation: 2015.* Department for Communities and Local Government, 2015.

4. Das-Munshi J, Bécares L, Boydell JE, Dewey ME, Morgan C, Stansfeld SA, et al. Ethnic density as a buffer for psychotic experiences: Findings from a national survey (EMPIRIC). *Br J Psychiatry* 2012; **201**(4): 282-90.

5. Bailey N, Livingston M. Population turnover and area deprivation: Policy Press; 2007.

6. Evans J, Middleton N, Gunnell D. Social fragmentation, severe mental illness and suicide. *Soc Psychiatry Psychiatr Epidemiol* 2004; **39**(3): 165-70.

7. Daniels MJ, Dominici F, Samet JM, Zeger SL. Estimating particulate matter-mortality dose-response curves and threshold levels: an analysis of daily time-series for the 20 largest US cities. *Am J Epidemiol* 2000; **152**(5): 397-406.

8. Mayor of London. *Air pollution monitoring data in London: 2016 to 2020.* Greater London Authority, 2020.

9. Eurostat. Urban population exposure to air pollution by particulate matter Luxembourg: Statistical Office of the European Union; 2020 [Available from: <https://ec.europa.eu/eurostat/web/products-datasets/-/T2020_RN210>.]

10. Krzyzanowski M, Cohen A. Update of WHO air quality guidelines. *Air Qual Atmos Health* 2008; **1**(1): 7-13.
